# Experimental and molecular dynamics studies of an ultra-fast sequential hydrogen plasma process for fabricating phosphorene-based sensors

**DOI:** 10.1038/s41598-021-95463-z

**Published:** 2021-08-09

**Authors:** M. Rajabali, H. Asgharyan, V. Fadaei Naeini, A. Boudaghi, B. Zabihi, M. Foroutan, S. Mohajerzadeh

**Affiliations:** 1 Thin Film and Nanoelectronic Lab, School of Electrical and Computer Engineering, University of Tehran, Tehran, Iran; 2 Division of Machine Elements, Luleå University of Technology, 97187 Luleå, Sweden; 3 Kish International Campus, University of Tehran, Tehran, Iran; 4 Department of Analytical Chemistry, School of Chemistry, College of Science, University of Tehran, Tehran, Iran; 5 Department of Physical Chemistry, School of Chemistry, College of Science, University of Tehran, Tehran, Iran

**Keywords:** Two-dimensional materials, Synthesis and processing, Nanosensors

## Abstract

Low concentration phosphorene-based sensors have been fabricated using a facile and ultra-fast process which is based on an exfoliation-free sequential hydrogen plasma treatment to convert the amorphous phosphorus thin film into mono- or few-layered phosphorene sheets. These sheets have been realized directly on silicon substrates followed by the fabrication of field-effect transistors showing the low leakage current and reasonable mobility for the nano-sensors. Being capable of covering the whole surface of the silicon substrate, red phosphorus (RP) coated substrate has been employed to achieve large area phosphorene sheets. Unlike the available techniques including mechanical exfoliation, there is no need for any exfoliation and/or transfer step which is significant progress in shortening the device fabrication procedure. These phosphorene sheets have been examined using transmission electron microscopy (TEM), Scanning electron microscopy (SEM), Raman spectroscopy and atomic-force microscopy (AFM). Electrical output in different states of the crystallization as well as its correlation with the test parameters have been also extensively used to examine the evolution of the phosphorene sheets. By utilizing the fabricated devices, the sensitivity of the phosphorene based-field effect transistors to the soluble L-Cysteine in low concentrations has been studied by measuring the FET response to the different concentrations. At a gate voltage of − 2.5 V, the range of 0.07 to 0.60 mg/ml of the L-Cysteine has been distinguishably detected presenting a gate-controlled sensor for a low-concentration solution. A reactive molecular dynamics simulation has been also performed to track the details of this plasma-based crystallization. The obtained results showed that the imparted energy from hydrogen plasma resulted in a phase transition from a system containing red phosphorus atoms to the crystal one. Interestingly and according to the simulation results, there is a directional preference of crystal growth as the crystalline domains are being formed and RP atoms are more likely to re-locate in armchair than in zigzag direction.

## Introduction

The evolution of two-dimensional (2D) materials has paved a new venue towards high performance electrical and optoelectronic devices where their application in bio- and opto-electronic devices and sensors as well as energy storing devices has drawn great attention^1–6^. Although graphene has been a successful 2D material, it possesses zero bandgap limiting its application as a semiconductor basis. To circumvent this deficiency, people have addressed other materials as transition metal di and tri-chalcogenides and some elemental nanostructures as phosphorene, borophene, silicene and germanene, etc. Among the elemental 2D nanosheets, phosphorene seems to be prominent owing to its unique catalytical^7^, sensing ^8^ electrical^9^ and optical properties. The most important of which is the presence of a direct bandgap at all levels of layers. While monolayers of phosphorene show a bandgap of 2.1 eV, this value drops to 0.3 eV for bulk (many-layers) form, called black phosphorus, yet it remains direct gap^10–12^.

Formation of black phosphorus has been a challenge over the past few years. High pressure and high temperature procedures^13^, sonication-based methods^14^ and even the phase transition using the presence of SnI_4_/Sn^15^ are the most common techniques employed to realize few- or monolayers of phosphorene through a complete phase transition from red phosphorus (RP) to black phosphorus (BP). Mechanical exfoliation of phosphorene flakes and their transfer to desired substrates is the main line of research for the fabrication of phosphorene-based nano-devices. Owing to the unstable and sensitive nature of phosphorene, its successful transfer onto the target substrate is a challenge and requires extreme precautions to avoid extended exposure to air and humidity^16–20^. Moreover, all these methods require formation of black phosphorus and its transfer to the desired substrates using mechanical or solution-based exfoliation. On the other hand, a bottom-up and direct formation of highly crystalline phosphorene sheets, including CVD, has been a challenge^21^.

Here, we have demonstrated a novel and ultra-fast technique for the formation of large area sheets of mono or few-layer phosphorene directly on a silicon wafer. Apart from a direct allotrope transformation on the target substrate, bypassing the exfoliation and transfer steps as two most challenging steps of the device fabrication, is another interesting advantage of this technique. The process is based on the conversion of the deposited amorphous RP layer into highly crystalline phosphorene sheets directly on the final substrates using a programmable sequential hydrogen plasma treatment. The required time for this phase transition is less than 2 h while the process temperature is maximum 350 °C. The transferred energy from hydrogen ion bombardment would lead to a combination of physical and chemical reactions which removes the topper layers (thinning) while the remaining part turns into a highly crystallized sheet with one or few layers (crystallization). The evolution of such large area layers has been investigated using characterization analyses including SEM, TEM, AFM and Raman spectroscopy. The results indicate a straightforward strategy to realize high quality 2D structures opening a new avenue to phosphorene-based (opto)electronic devices and sensors. Considering the extensive studies dedicated to the interaction of small molecules and phosphorene^22^, after completing crystallization, a phosphorene-based transistor was fabricated showing a mobility value of 105 cm^2^/Vs and the I_on_/I_off_ switching ratio of 10^2^ and eventually, to employ it as a practical device, we investigated its sensitivity to different concentrations of L-Cysteine (L-Cys). L-Cys (2-amino-3-sulfhydryl propanoic acid) is one of the amino acids with the main characteristic of the thiol or sulfur (-SH) group. The thiol functional group in L-Cys plays an important role in the biological activities of proteins and enzymes. Recently, the detoxifying properties and safety resistance of this material have been proven. L-Cys plays a key role in diagnosing various diseases such as Parkinson's, amnesia, etc.^23^. The amount of this substance in the human body is essential and is one of the important biomarkers which its inadequacy causes slowed growth in children, depigmentation of hair, edema, lethargy, loss of muscle and fat, skin injuries, and weakness^24^. This amino acid is not made in the human body and is obtained with proper nutrition and L-Cys-containing elements should be present in the diet of individuals. Accordingly, L-Cys measurement is of a great importance in human body fluids such as blood or urine, as well as their food^25^.

Furthermore, and in one step forward towards understanding the transition circumstance from an entirely amorphous structure to a crystalline sheet, a simulation has been carried out using reactive molecular dynamics (MD). There have been several reports on thermal^26^ and mechanical properties^27,28^ of black phosphorus and also the characteristics of phosphorene nanotubes^29^, but to the best of our knowledge, there is lack of enough study on presenting how black phosphorus is synthesized from amorphous red phosphorus. Recently, Shriber et al. explained catalyst based formation of black phosphorus using density functional theory^30^ and some studies have been performed on presenting the mechanism of exfoliation^31^ but there is no trace of a thorough simulation study accompanied with a comprehensive experimental support. In our present work, reactive molecular dynamics simulations have been performed to investigate the direct crystallization of red phosphorus through employing energy from the hydrogen bombardment with importing data provided by experimental section to become as close as possible to real circumstances. After defining the initial system and applying the hydrogen bombardment, the reactive molecular dynamics simulations at different states of crystallization have been performed to present the evolution of this allotrope transformation.

## Experimental section

**Figure 1:**
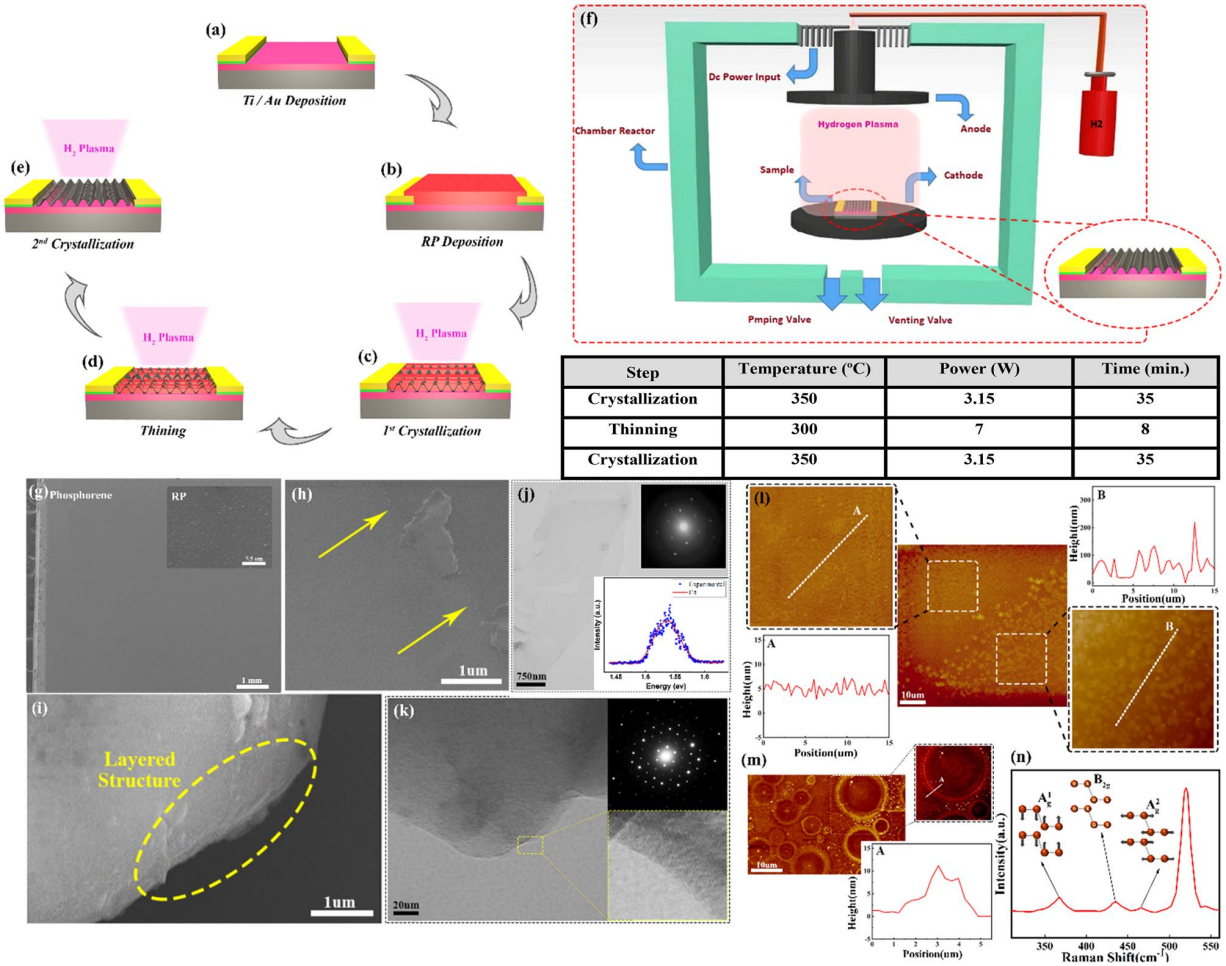
The synthesis process and material characterization: (**a-e**) Evolution of few-layer phosphorene sheets demonstrating the crystallization trend of the formation of a thoroughly crystalline layers: (**a**) Pre-lithography, (**b**) Initial amorphous layer, (**c**) first crystallization, (**d**) Thinning and (**e**) the second crystallization steps. (**f**) The schematic image of dc_PECVD as well as test parameters of the sequential hydrogen plasma treatment. (**g**) A low-magnification SEM image of phosphorene sheet grown on a silicon substrate, illustrating an ultra-large area with no sign of stacked or bulk inclusion. The inset shows the deposited RP layer before the plasma treatment. (**h**) An SEM image of the edge of a sample corroborating the conformal and ultra-thin phosphorene layer on a silicon substrate. (**i**) An SEM image of the edge of a thick phosphorene layers indicating layered structure of the synthesized sheets. (**j**) An ultra-large mono-layer phosphorene. The top inset shows a clear diffraction pattern confirming the formation a crystal layer. The bottom inset confirms the existence of monolayer phosphorene through the photoluminescence analysis. (**k**) Bright field and high-resolution TEM image beside the corresponding diffraction pattern of a few-layer phosphorene sheet. (**l**) A comparison between AFM micrographs of the surface roughness of the sample (A) after and (B) before the sequential plasma treatment. (**m**) AFM micrograph from the surface degradation of a phosphorene layer appeared after one-day exposure to the ambient situation. (**n**) Raman spectrum of a highly crystalline few-layer phosphorene sheet.

Initially, we need to prepare the suitable substrate to start the crystallization process. As stated, this novel technique intends to bypass the exfoliation and transfer during the device fabrication. To fulfill this purpose, a pre-lithography has been investigated on the substrate which is supposed to bear the final phosphorene layer. After cleaning a Si/SiO_2_ substrate and rinsing in deionized (DI) water, a radio frequency (13.56 MHz) sputtering unit has been utilized to deposit a Ti(5 nm)/Au(50 nm) layer. Next, the desired pattern which is an interdigital structure with 10 μm separation between digits are achieved by means of a lift-off method (Fig. 1a). Deposition of an initial red phosphorus layer is the first step of the crystallization, leading to the formation of an amorphous layer directly on silicon substrates. As in our previous work^32^, 0.1 g of RP powder (Merck ≥ 97%, Hohenbrunn, Germany) with a handy-press apparatus is needed to prepare a RP tablet. Silicon substrates and RP tablet are placed in a plasma reactor (dc-PECVD) to deposit the desired layer onto substrates. The tablet and samples are in the hot and cold zones of the plasma reactor, respectively. The chamber is evacuated using a rotary vane pump. When the base pressure reaches to 10 mtorr, the hot section of the reactor is adjusted to warm up to a temperature of 450 °C. A simultaneous flow of hydrogen carrier gas (20 sccm) for a period of 45 min will lead to the deposition of the desired RP layer on the silicon wafer. Obviously, the final thickness is directly related to the selected gas flow, time, and the number of tablets. To realize a uniform phosphorene layer, after achieving the conformal layer of RP which is deposited on the silicon substrate (Fig. 1b), the plasma reactor is exploited again but this time as the source of external energy for the transformation of the amorphous allotrope into the crystalline one. Accordingly, a sequential hydrogen (H_2_) plasma process has been developed including three sequences: initial crystallization, thinning and final crystallization. In all sequences, the RP-coated substrate is placed on the lower plate (cathode) of two parallel stainless-steel electrodes and the defined parameters including temperature of the reactor, plasma power, type, and flow of the carrier gas, are then set for each step. At first, hydrogen carrier gas with a flow rate of 10 sccm is introduced to the chamber to smoothly elevate the temperature throughout the quartz tube and then along the first sequence, so called 1st crystallization, a 35-min plasma treatment is applied with a dc power of 4 Watt at a moderate temperature of 350 °C. In initial moments when the possibility of evaporation compared to crystallization is more likely to happen, a preliminary thinning occurs but most of the imparted energy has been devoted to crystallizing the layer and as the time lapses, a denser and more stable layer will be formed. We assume that this reconfiguration in atomic arrangement, which is called crystallization, is due to impingement of the present reactive species in plasma on the surface of the sample. In this sequence, the interactions between the phosphorus atoms and the hydrogen ions and radicals result in the diffusion of hydrogen atoms into the amorphous matrix followed by the formation of intermediate bond-centered P–H–P configurations. Since the state is not energetically stable, a disorder-to-order transition would happen, and the system turns to further relaxation of P-P bonds. The final phosphorene grains synthesized in this sequence, as can be seen in Fig. 1c, are conducted by this bond reformation and eventually a network rearrangement^33^.

Employing the combination of crystallization and thinning instead of a thorough crystallization step would lead to bypassing the exfoliation step which is a progress towards facilitating the device fabrication. Since the process is improving, the evaporation would be less probable, the second sequence would be faster in time but stronger in power to perform a secondary thinning before the realization of the final phosphorene layer (Fig. 1d). The process is followed by employing the crystallization parameters for the second time, assuring the phase transition proceeds all over the surface (Fig. 1e). Once the last step is completed, the reactor is cooled down to room temperature and the sample is unloaded for further characterization analyses and device fabrication. Part f of Fig. 1 depicts the schematic image of the dc-PECVD as well as the parameters used in each sequence for the plasma treatment.

To pursue the evolution of these phosphorene sheets, several characterization analyses including field emission scanning electron microscopy (FE-SEM), atomic-force microscopy (AFM), transmission electron microscopy (TEM) and Raman spectroscopy have been performed. Electron microscopy analyses (SEM and TEM) are the first and main tools to study the evolution of the crystallization of the amorphous RP layer and the transition to crystalline sheets. The first indication of changes was apparent in SEM figures. The SEM images have been taken using an electron microscopy apparatus (Hitachi 4160, FE-SEM) to study the morphology of the layers. Figure 1g illustrates a top view SEM image at low magnification indicating a major part of the surface of the sample after completing the sequential plasma treatment. As the first promising observable clue, a large surface with no sign of contamination and stacked or thick separated flakes, with an obvious difference compared to RP layer (the inset) has been obtained. The other indication of a fundamental transition is the layer stability under extended exposure with a rather high-energy electron beam in SEM machine. According to the intrinsic instability of an amorphous layer, the presence of RP parts in the sample would lead to bubble and rupture. As stated before and thanks to the direct crystallization on the initial substrate, there is no specific borders and it would be an advantage not to limit the user to fabricate the further devices in a defined area; unlike the mechanical exfoliation and the routine techniques. Although uncommon, but Fig. 1h displays the edge of a sample corroborating the conformal and ultra-thin phosphorene layer on a silicon substrate. Thick parts, rarely observed in the samples, can be informative to investigate the structure of layers. Figure 1i displays the edge of one of these infrequent thick layers. As shown in the figure, the layered structure of the flake is evident.

To examine the crystalline quality of the synthesized layer, a transmission electron microscope (Philips CM300 TEM operating at 200 kV) was used. The samples bearing large area phosphorene sheets and prepared by sequential hydrogen plasma, have been used to be evaluated. The sheets are transformed on a carbon grid and the have been studied. A highly crystalline monolayer phosphorene sheet has been observed originating from the deposition of an initially thinner layer. As seen in Fig. 1j, an ultra-large sheet with a clear SAED pattern (the top inset) is illustrated which ascertains a complete phase transition with no evidence of amorphous inclusion. To confirm the layer number, photoluminescence (PL) spectroscopy analysis using a 405 nm laser has been conducted. The bottom inset in Fig. 1j shows the measured peak is at 1.53 eV corroborating the presence of monolayer phosphorene sheets consistent with the previous reports^34^. Part (k) of Fig. 1, including three parts of bright field, selected area electron diffraction (SAED) pattern and high-resolution TEM (HR-TEM) images, affirms the morphology and nature of the sheets. Moreover, Fig. 1k presents the layered structure of the synthesized few-layer sheets corroborating the thorough transition from the amorphous RP layer to layered crystalline sheets. The observed SAED pattern (top right) also leads to the same consequence by emerging the bright and sharp spots with a significant intensity expressing the high quality crystalline layer. HR-TEM figure (bottom right) indicates a magnified part of the figure, whereas an atomic fringe pattern with a lattice distance of ~ 0.26 nm can be observed for the phosphorene sheet. Consequently, through a treatment by choosing the optimum parameters, few- and even mono-layer phosphorene sheets are realized using the sequential hydrogen plasma.

The AFM analysis is the next step to study the morphology and other appeared changes compared to the original state. An inspection of the difference between the surface roughness before and after plasma treatment would be an informative comparison. For this purpose, half of the surface of one sample has been covered before applying the sequential hydrogen plasma, while the second half is exposed. After the procedure, the interface has been investigated using an NT-MDT AFM apparatus in non-contact mode with an NSG10 tip (~ 190 KHZ resonance frequency).

As seen in part (l) of Fig. 1, the achieved micrograph shows its surface roughness in two different regions. The magnified image and the corresponding height profile of a phosphorene sheet and an annealed RP layer are demonstrated in parts A and B, respectively. Furthermore, an observation of the surface degradation after exposing to the ambient conditions can be a significant evidence for the presence of phosphorene layers. Consequently, the appearance of bubbles after one-day exposure to air without any surface passivation, would be another indication of the existence of phosphorene layers. The main reason behind the initiation and further expansion of the surface degradation is assumed to be the presence of itinerant vacancies accumulated either at the edges or the grain boundaries (possibly where the smaller grains are merging to form large area sheets). Oxygen molecules can be trapped at these defect sites 5000 faster than a perfect site within the layer and eventually lead to oxidation and further degradation^35^. Figure 1m, displays bubbles in different sizes spreading all over the substrate. The magnified image represents one of these bubbles and the corresponding height profile taken along the edge of exploded surface. The measured height from an exploded feature is more than the thickness of the phosphorene layer. Accordingly, the estimated thickness of the initial sheets is less than 10 nm.

Raman spectroscopy has been employed as the last characterization analysis to precisely examine the crystalline quality of phosphorene sheets. The investigation has been carried out using a Teksan-Opus Raman microscope with a Nd:YAG green laser (λ = 532 nm) and as seen in Fig. 1n, a complete phase transition from an amorphous layer to a thoroughly crystal structure has proceeded. Emerging all three characteristic peaks located at 362 cm^−1^, 437 cm^−1^ and 466 cm^−1^, beside the standard first order peak of the silicon substrate in 520 cm^−1^, elucidates the crystal nature of synthesized sheets. As displayed atomically in Fig. 1n, these three peaks are attributed to one out-of-plane peak, A^1^_g_ and two in-plane vibration modes, B_2g_ and A^2^_g_, respectively. One can see that Raman frequencies are in good agreement with the previous reports and revalidate this claim which plasma treatment does not lead to substantial structural defects within phosphorene layers and accordingly, the final synthesized layers do not reveal any sign of either the existing strain or any other discrepancy compared to the crystalline sheets^36,37^.

**Figure 2:**
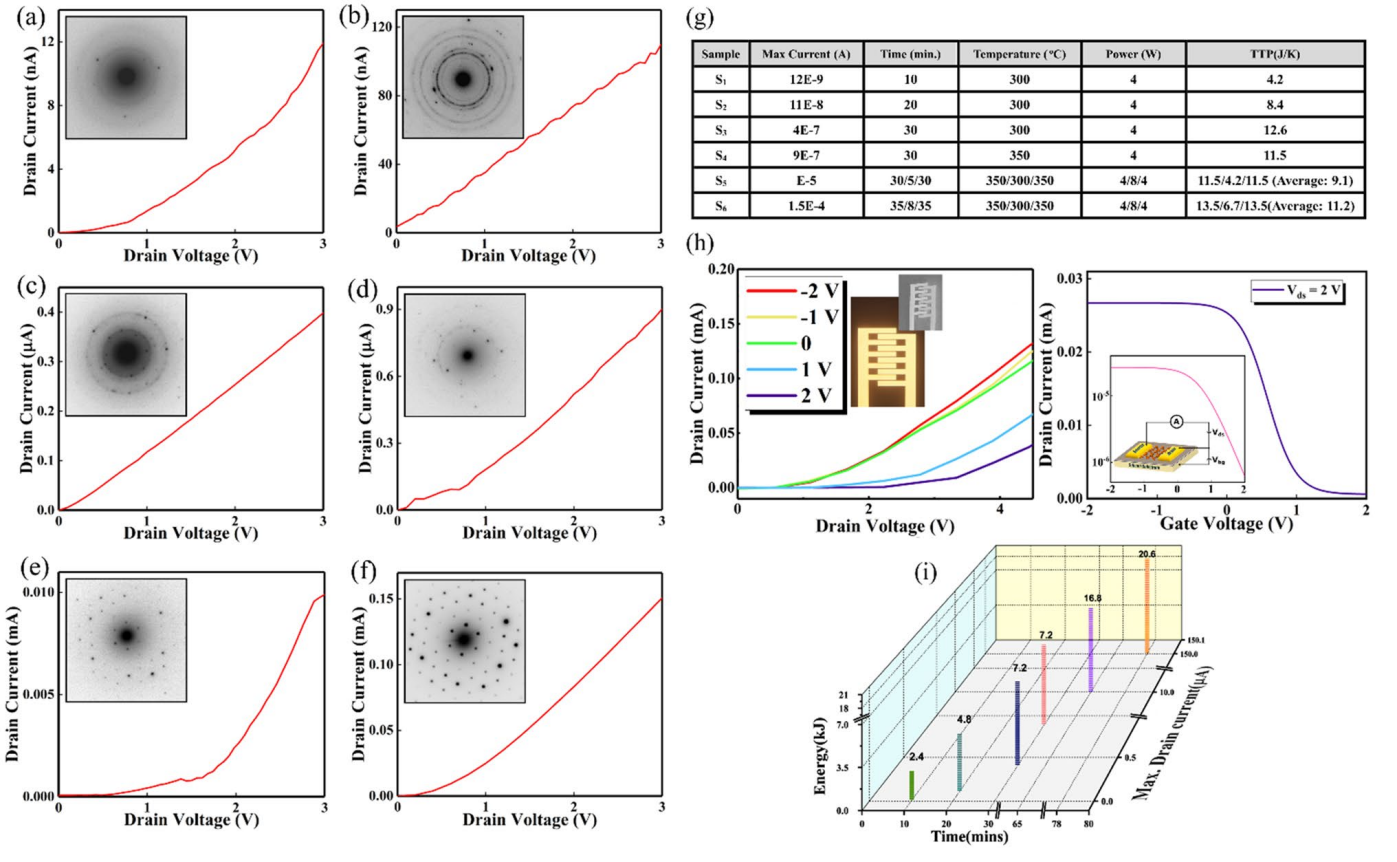
Electrical characteristics of phosphorene sheets: (**a-f**) Recorded currents of six different phosphorene-based resistors for different states of the crystallinity of synthesized sheets; from a partial crystal sample to a highly crystalline one. (**g**) Sample parameters of the different hydrogen plasma treatments. (**h**) Left: The output characteristic of a phosphorene-based field-effect transistor. Right: The linear and semi-log transfer curves. (**i**) A comprehensive correlation between the recorded current and the different process parameters (time and imparted energy).

To elaborate the priority of a sequential crystallization process, we next aimed for describing different steps paved to set the desired sequential parameters. Time, temperature, and power are three determinative factors governing the quality of the final layer. To scrutinize the evolution of few-layer phosphorene sheets while changing the test parameters, an intensive electrical characterization has been done on samples in different crystallization status. For these measurements, p-type silicon substrates with a resistivity of 1-Ωcm and a thermal oxide as the gate insulator (300 nm) have been used. The IV characteristics of six different resistors fabricating on phosphorene layers with six different states of crystallinity are presented in Fig. 2a–f. The insets are the diffraction patterns corresponding to each phosphorene channel. In all graphs, the drain-source voltage (V_ds_) has swept from 0 to 3 V. Similar gold deposition and preparation steps are applied for all samples. As the phase transition progresses, the measured current of the phosphorene-based resistors has been enhanced; starting from a value of 12 nA for the first stage attributed to a sample with the most amorphous parts to a high value of 0.15 mA which is for a highly crystalline sample. Furthermore, by improving the crystalline quality of the layer, the bright spots have been emerged in diffraction patterns. The illustrated table in Fig. 2g describes all samples (S_i_) and their relevant parameters considered to achieve the desired one (i.e. Fig. 2f). To compare the input values in each test, a new parameter so-called TTP has been introduced using the following formula:

1 $$ TTP_{i} = \frac{{T_{i} \times P_{i} }}{{TE_{i} }} $$

where *T*_*i*_, *TE*_*i*_ and *P*_*i*_ are input time, temperature, and the dc power, respectively. Initially, a single-sequence experiment has been chosen (*S*_*1*_). Furthermore, time was increased in next two samples (*S*_*2*_ and *S*_*3*_) leading to an increment in recorder current. It is worth mentioning that any enhancement in plasma power resulted in a precise deterioration of phosphorene layer and further failure in recording current. Then, by elevating the temperature, higher output current has been achieved (*S*_*4*_). At this point and in order to involve all three parameters in a gradually increasing manner, the innovative sequential hydrogen plasma was introduced. It not only crystallizes the RP layer with an appropriate treatment but forms thin phosphorene layers with no need to any further exfoliation by employing an intermediate thinning step (*S*_*5*_ and *S*_*6*_). The last sample (*S*_*6*_) demonstrates the maximum recorded current which is the result of completion of the phase transition. More increment in imparted energy would lead to deterioration and further sputtering of the synthesized phosphorene layer. It should be noted that the last column is dedicated to measured TTP indicating by an increment in heat capacity in each step, more energy out of the required amount for a complete crystallization is provided leading to a higher recorded current.

Next, the electrical measurement of a field effect transistor fabricated on the phosphorene sheets with optimum parameters has been carried out. The output characteristic and the corresponding transfer curve of phosphorene-based FET is plotted in left and right side of Fig. 2h, respectively. The output characteristic is extracted by sweeping I_ds_ versus V_ds_ ranging from 0 V to 4.5 V with low back-gate voltage (V_g_) ranging from − 2 to 2 V with a step of 1 V. The explicit decrease in recorded current with an increase in V_g_, confirms a p-type semiconducting behavior of the transistor. The inset in left-sided image illustrates the optical and SEM image of the interdigital pattern used for RP deposition and further device fabrication. There is also a schematic image of the fabricated FET including source and drain contacts as well as the phosphorene layer as the channel (right-sided image). To calculate the mobility, the transfer characteristic in linear and semi-log (inset) scales have been considered where the gate voltage has swept from −2 to 2 V at V_ds_ of 2 V. By extracting the data from the linear transfer curve, the hole mobility value and the switching ratio (I_on_/I_off_) can be obtained. The carrier mobility is calculated using the following statement:

2 $$ \mu_{FE} = \frac{L}{W}\frac{1}{{C_{g} V_{ds} }}\frac{{dI_{ds} }}{{dV_{g} }} $$

where *L* and *W* are the channel length and width, respectively, *C*_*g*_ is the capacitance per unit area and *dI*_*ds*_*/dV*_*g*_ represents the maximum slope of the linear transfer curve. In this case, a mobility of 105 cm^2^/Vs and an I_on_/I_off_ ratio of 10^2^ at room temperature have been achieved. This value corroborates the effectiveness of the sequential growth procedure for further fabricated devices and sensors.

Eventually and to demonstrate the correlation between the recorded current obtained in electrical tests and the employed process parameter i.e., time and energy, a 3D-graph (part (i) of Fig. 2) is illustrated within the recorded current is an indirect representation of different steps of crystallization correlating with the test conditions. As the imparted energy and time increase, the current will enhance indicating the evolution of phosphorene sheets.

One of the most important advantages of this ultra-fast, reliable, and reproducible process is its potential to be mass-produced and used as sensors and detector devices. Direct conversion of the phosphorene from amorphous red phosphorus from one side and eliminating the transfer step from the other side turned this technique potentially appropriate for the mass production of phosphorene layers. Moreover, the overall time of preparing a sample is less than two hours and it is possible to simultaneously load and process more than one sample. Even if one desires, two parallel stainless-steel electrodes and chamber size can be customized to be able to bear bigger substrates.

**Figure 3:**
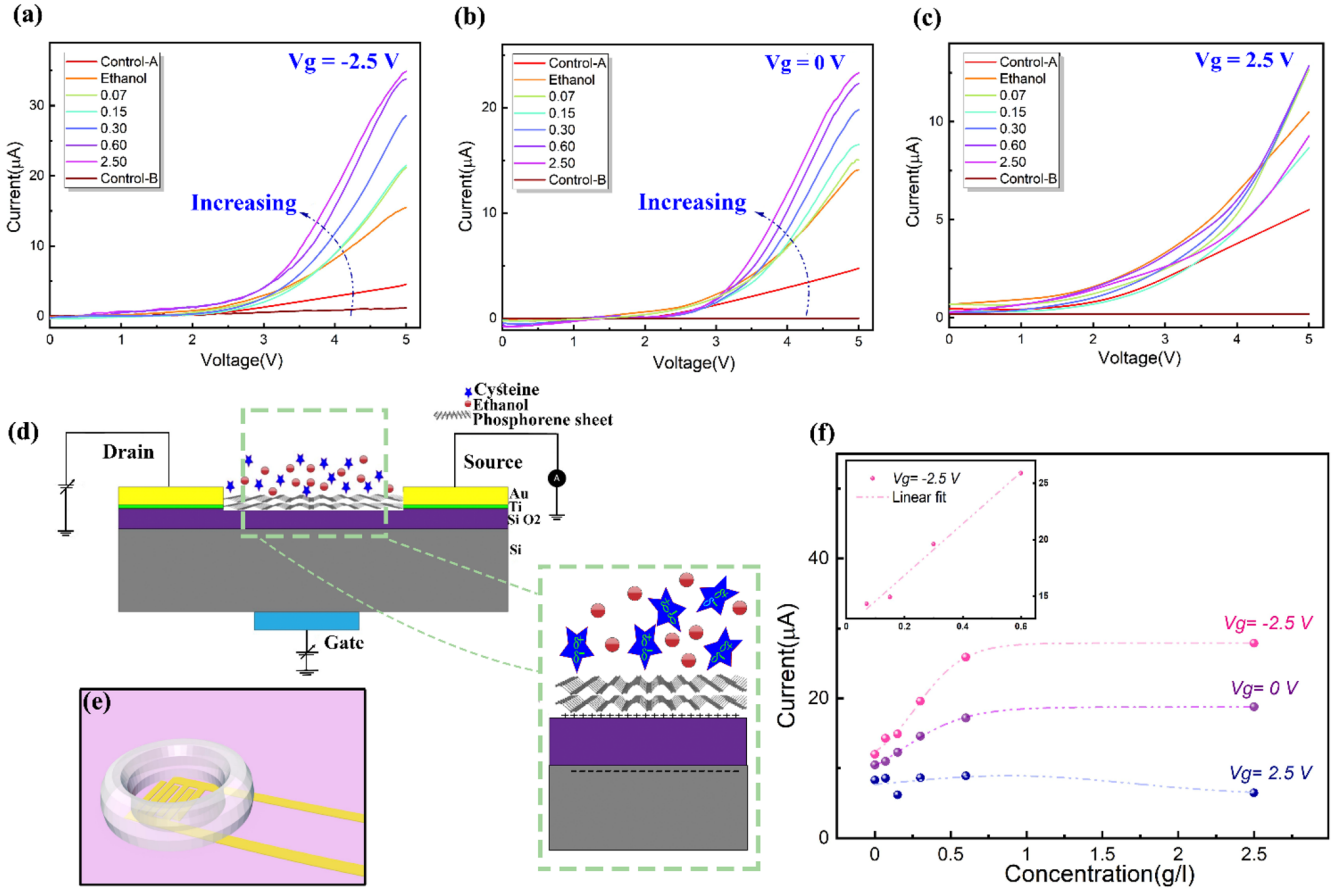
Low concentration L-Cysteine sensing by phosphorene-based FET: (**a-c**) Recorded output characteristic of a phosphorene-based field-effect transistor at different gate voltages for various concentrations of L-Cys in ethanol. (**d**) A schematic illustration of the employed sensing transistor. The magnified image illustrates the intercorrelations of soluble components with the surface of the FET channel. (**e**) A schematic image which illustrates a 3D-view of the employed setup for the detection of different concentrations of L-Cys. (**f**) The calibration curve at a drain-source voltage of 4.5 Volts while the inset shows the calibration curve for the gate voltage at − 2.5 Volts.

Accordingly, and to study the potential of this plasma-assisted phosphorene-based FET, the electrical characterization of different devices bearing solutions of different concentrations of L-Cys has been performed. To explore the effect of the V_g_ and further induced charge on the electrical responses, the output characteristic was recorded by sweeping V_ds_ from 0 V to + 5 V at various gate voltages of − 2.5 V, 0 and 2.5 V. Figure 3a–c reflects the findings from applying negative, zero-biased, and positive V_g_, respectively, comparing the sensitivity of the device at different applied the back-gate voltage. Furthermore, Fig. 3d illustrates the intercorrelations of soluble components with the surface of the FET channel. L-Cys molecule has some atoms with different electronegativities (in comparison with the side atoms) and it also has non-bonding pair electrons on heteroatoms. Accordingly, L-Cys has a partial negative charge (${\delta }^{-}$) on its heteroatoms^24^. By applying a negative V_g_, electrostatic attraction between the surface and L-Cys from nucleophilic heteroatoms, bearing a partial negative charge (${\delta }^{-}$), results in approaching the molecule to the surface and eventually, the carrier concentration within the conducting channel is changed leading to a measurable increment in the recorded current between the drain and the source. Interestingly, the same measurement after employing a positive voltage indicates the findings in contrary to the previous results. By applying a positive V_g_, the negative voltage induced on the surface causes the L-Cys molecules to be driven away from surface. This will lead to an unstable and irregular behavior. It is apparent that at any constant V_ds_ and compared with the negative gate voltage, the zero-biased gate has a lower (~ 10%) I_ds_ value. A possible explanation for this might be the weak interaction of L-Cys with surface which reduces the conductivity. A 3D-Schematic image has been also provided to present how the interdigital pattern is surrounded by a well bearing the solution (part (e) of the Fig. 3).

To picture the sensitivity regime of the fabricated devices, as shown in Fig. 3f, I_ds_ in terms of different concentrations of L-Cys (the calibration curve)​​ has been displayed at Vg of + 2.5, 0 and − 2.5 V (at a fixed V_ds_ of 4.5 V). By considering their behavior, one can interpret the value of − 2.5 V as the most effective set V_g_ due to its higher sensitivity. Since the channel is an ultra-thin few-layer phosphorene, increasing the L-Cys ​​concentration beyond 0.6 mg/ml would saturate the channel and the surface conductance is no longer affected by the concentration of L-Cys, hence and as can be seen in the inset, the dynamic linear range for measuring from 0.07 to 0.6 mg/ml with a regression coefficient of 0.982 has been obtained. The measurement is performed at V_ds_ + 4.5 V because at this certain value, the highest sensitivity for measurement is obtained ($12.3\mu A/\left(g/l\right)$). It is worth mentioning that the recursive behavior of the current and recording the relatively same current in Control-B (after removing L-Cys) as in Control-A (before applying L-Cys) supports the idea that during the measurement, no damage has occurred to the phosphorene structure.

As stated before, a thoroughly bottom-up technique has been introduced with no need to any exfoliation and transfer steps. This technique would be a new, ultra-fast and high-yield growth technique especially in electrical and bioelectrical applications including sensing the low concentration of L-Cys solution with no trace of degrading during the measurement.

## Simulation section

In this part, the details of the reactive molecular dynamics simulations are presented. It is worth noting that a system was designed to assess the formation process of phosphorene from the initial structure of red phosphorus. Moreover, a distinct system was design and simulated to examine the directional preference in crystal growth and the modeling and simulation details of each system are presented separately.

### System specifications and simulation method: Crystallization procedure

**Figure 4:**
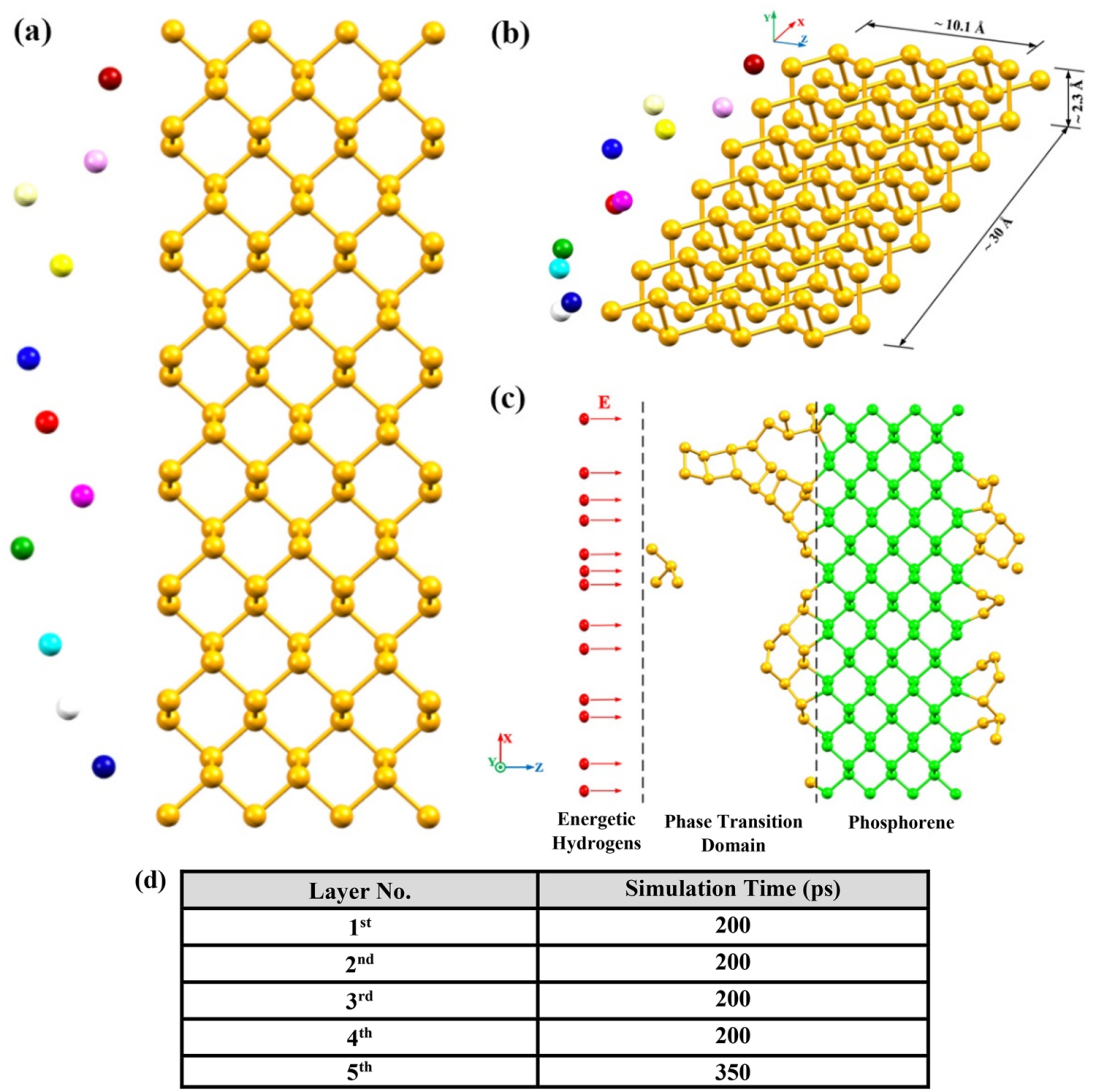
Initial configuration of the system: (**a**) The front view of the system in the XZ plane. (**b**) The 3D view of the system of study along with the schematic dimensions of the crystal structure of the black phosphorus seed. The snapshots were rendered by Mercury^40^. (**c**) High-energy hydrogen ions colliding with the RP zone. The snapshots were captured by Mercury^40^. (**d**) Simulation time for different steps of adding RP segments.

Initially, a single-layer of black phosphorus crystal with (30 × 2.3 × 10.1) Å^3^ dimension including 196 phosphorus atoms was produced to investigate the crystallization procedure. To create the amorphous structure of red phosphorus, an initial block of white phosphorus was primarily heated using MD simulation. the phosphorus block was equilibrated at 620 K under NVT ensemble to form the initial amorphous structure for further simulation of the crystallization procedure^38^. Since the accessible time scale in MD simulation differs from the actual one in experimental process, if a bulk fraction of RP is placed in the simulation box, even in partial mode, the desired phase transformation is most likely not observable. Accordingly, in MD simulation of crystallization procedure, a segment of single-layer black phosphorus (phosphorene) is added to the simulation box providing a nucleation site towards facilitating the crystal growth; meanwhile decreasing the required time for the final complete crystallization. Consequently, the bulk structure of red phosphorus is divided into smaller segments to be added layer by layer at the beginning of each stage of the simulation. As seen in Fig. 4a, a segment of red phosphorus structure, containing 11 phosphorus atoms, is placed at a certain distance of the black phosphorus crystal and simulated under the NVT ensemble. The resulting structure is thereafter subjected to the impact of high-energy hydrogen ions under the microcanonical ensemble. Based on the designed mechanism for bombarding the mixed matrix of RP and BP structures, they are exposed to the energetic impact of a hydrogen atom per simulation step. The ion beam energy is adjusted according to the energy of the hydrogen plasma proposed in the experimental section. To describe the atom type of energetic ions in the simulation scheme, a hydrogen ion is placed in the initial structure of the model inside the simulation box.

Once the bombarded structure of red phosphorus is completed, periodic boundary conditions are applied to the simulation box for series of MD simulations in the current investigation. Figure 4 reveals the different positions of the system of study, including the planar view in the XZ plane as well as the schematic 3D view in part (a) and (b), respectively. Moreover, the impact direction and the initial position of high-energy hydrogens relative to the RP structure are displayed in Fig. 4c. Simulation time for different steps of adding RP layers is provided with the table shown in part (d) of the image. During all stages of the simulation, the black phosphorus seeds were assumed to be fixed. MD simulations were carried out on a system using the molecular dynamics program LAMMPS^39^ with 1 fs as the time step. Finally, the system contains BP and RP blocks with the size of (33.6 × 5.9 × 19.3) Å^3^ in x, y, and z directions, respectively.

In order to provide desired conditions for the covalent bond formation/breaking and produce a new layer of the crystalline structure of BP from RP atoms during the simulation, a transferable reactive force field was carried out^41^. The overall system energy in reactive force field can be expressed using the following equation:

3 $$ E_{system} = E_{bond} + E_{over} + E_{val} + E_{60Cor} + E_{tors} + E_{\lg VdW} + E_{Coulomb} $$

In Eq. (3), *E*_*bond*_ represents the bond energies calculated from the corrected bond orders, *E*_*over*_ imposes an energy penalty on the system for an over coordinated atom. A term of energy (*E*_*val*_) is defined to take the energy contributions from the valence angle into consideration. The partial contribution for the energy of the torsion angle (*E*_*tors*_) and Coulomb interactions (*E*_*Coulomb*_), as well as the low-gradient van der Waals correction term (*E*_*lgvdW*_)^42^, are also included.

The phosphorus is commonly anticipated to prefer valence angles near 101°^43^. Consequently, the strain energy of bonds makes the P_4_ cluster unstable. This problem can be settled by taking the effect of d-orbitals into account^44^. An angle correction term (*E*_*60Cor*_) is added as a contribution of the overall energy to properly tackle the stability challenge of the P_4_ cluster^42,45,46^. Excluding the last two terms of overall energy, others can be expressed as a function of the bond order parameter.

Electrostatic interactions (*E*_*Coulomb*_) are computed between all atom pairs, in which the atomic charges are determined according to connectivity and geometry by Electron Equilibration Method (EEM)^47^. This feature allows reactive MD to describe charge transfer in chemical reactions^48^. In the current investigation, a developed version of reactive MD with parameters for phosphorus and hydrogen was employed providing a desirable description of the chemical interaction among RP structure, monolayer BP, and hydrogen plasma. MD simulations were carried out on three-dimensional space under the NVT ensemble and periodic boundary conditions were imposed on all dimensions of the box. During the crystallization process, the temperature of the system was maintained near the desired set point using the Nosé-Hoover thermostat^49^.

### System specifications and simulation method: directional preference in crystallization

**Figure 5:**
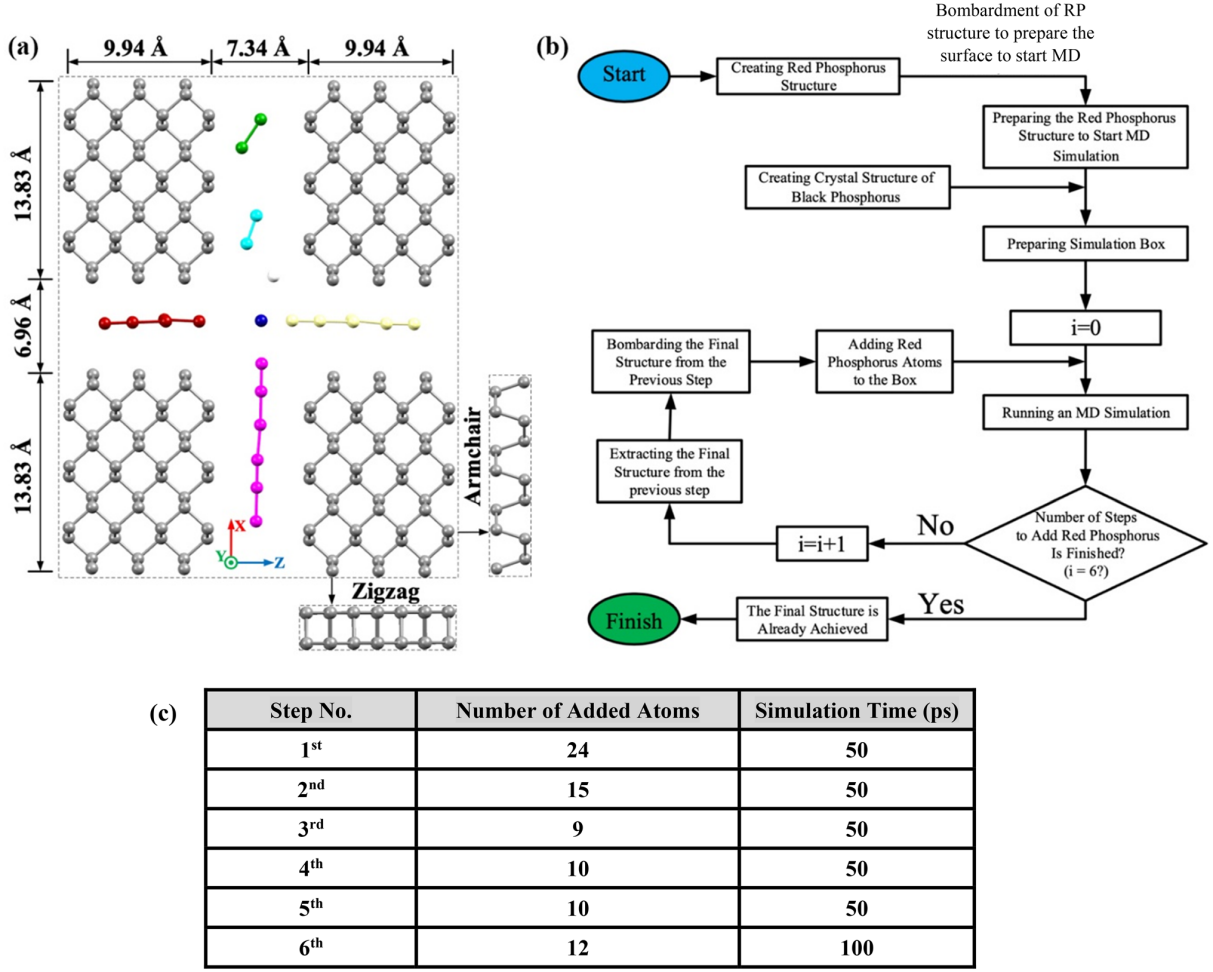
MD Simulation steps. (**a**) Initial arrangement of the system in the XZ plane to assess the directional preference during crystallization procedure. The side viewpoints of the BP blocks in zigzag and armchair directions are illustrated. The snapshots were rendered by Mercury^23^. (**b**) A schematic flowchart for the implementation of the modeling and simulation steps to investigate the directional preference in crystal growth. (**c**) Simulation details for different steps of adding RP atoms.

The second system of study is designed to examine the directional preference in the crystallization process. An initial structure file consisting of four BP blocks and 192 atoms with the dimensions demonstrated in Fig. 5a, was generated. In the MD simulation, the red phosphorous, prior to being placed in the simulation box to initialize the process of structural transition by nucleation, is exposed to hydrogen ions bombardment with the same energy level of plasma beam in the experimental section. Accordingly, a red phosphorus segment containing 24 atoms, which had been previously bombarded with high-energy hydrogen ions for 100 ps under the NVE ensemble, was added to the simulation box. The final configuration of the system to start MD simulation of RP and BP blocks is depicted in Fig. 5a.

It is worth mentioning that Eq. (4) was utilized to calculate the velocity of hydrogen ions in the initial bombardment process of the RP block:

4 $$ v = \sqrt {\frac{{2Eq_{e} }}{u}} $$

In this regard, *E* is assumed to be the energy of the ionic hydrogen beam in terms of eV, *q*_*e*_ denotes the elementary charge which is equal to 1.602 × 10^–19^ C and *u* is the proton’s mass (1.673 × 10^–27^ kg). The amount of energy of the ionic hydrogen beam during the simulation is properly in accordance with the energy of the hydrogen plasma described in the experimental part.

In next step and by fixing the BP blocks inside the box, the motion and arrangement of the RP atoms were examined for 50 ps under NVT ensemble while the dimensions of the simulation box were equal to (41.2 × 5.8 × 30.9) Å^3^ in X, Y, and Z directions, respectively. The Nosé-Hoover thermostat was also utilized to control the system temperature during the crystallization process^33^. Upon the simulation was accomplished at this stage, the overall structure of the phosphorus surface is exposed to the energetic impacts of hydrogen ions at the same velocity as the ions in the hydrogen plasma for 100 ps under the NVE ensemble. Subsequently, by removing the hydrogen ions from the system and adding new red phosphorus atoms to the box, the simulation under NVT will be performed and this cycle will proceed until the last step of adding RP atoms. For the sake of clarity, a schematic flowchart has been arranged to illustrate modeling and MD simulation steps (Fig. 5b).

Moreover, the embedded table in Fig. 5c displays the details of the steps to add red phosphorus atoms in the system. It has provided the simulation time for inserting RP atoms of each layer in crystal sites of BP along with the number of added atoms in each step.

### Investigating the process of crystal formation

According to the results of electrical characterization in the experimental part, the process of crystallization occurs as nucleation and expansion of crystalline sites.

**Figure 6:**
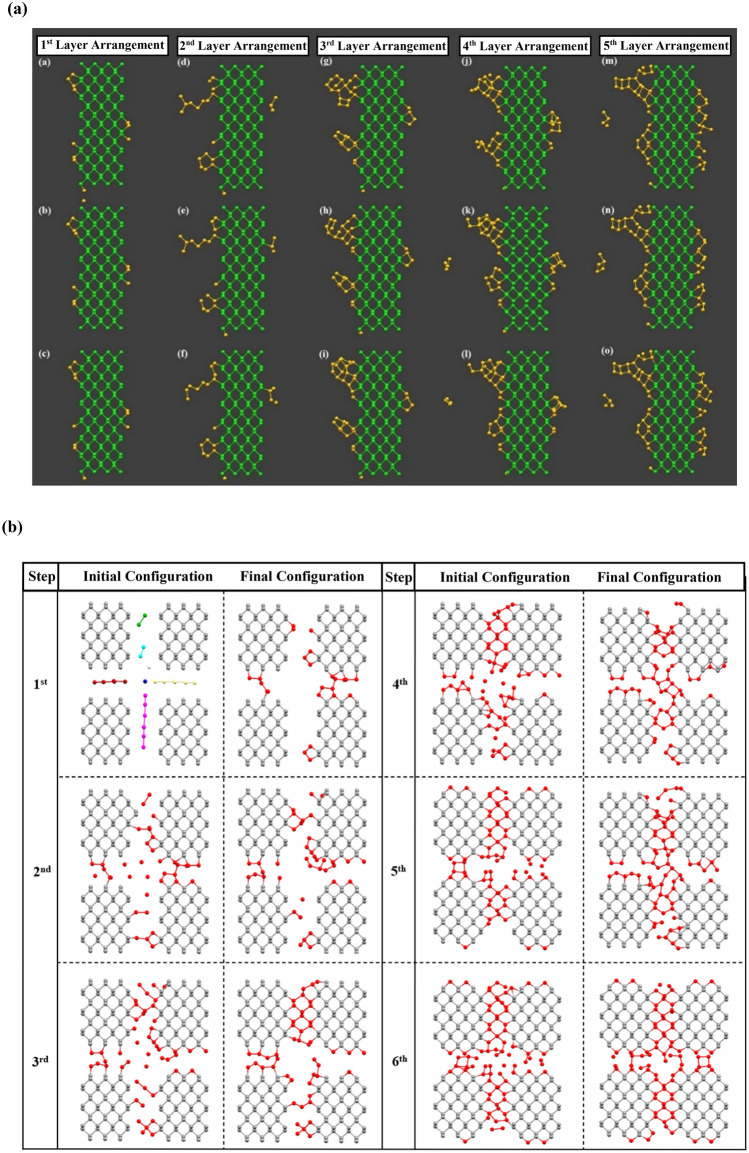
Arrangement of the red phosphorus atoms next to the phosphorene monolayer after adding each segment of red phosphorus. (**a**) From the first to fourth columns, each snapshot from top to bottom corresponds to 66.7 ps, 133.3 ps, and 200 ps, respectively. In the fifth column, each of the snapshots from top to bottom panels corresponds to 116, 232, and 350 ps, respectively. (**b**) The initial and final configurations of the system after adding the RP atoms in each step.

To review the previous section, as quickly and accurately as possible, an initial seed was inserted in the box to begin the MD simulation and make a detailed assessment of the phase transition process from an amorphous structure to the crystal one. Figure 6a displays the progressive formation of the covalent bonds between the RP atoms and the BP structure as well as the arrangement of RP atoms in the crystalline sites of the BP structure.

As perceived from Fig. 6a, the atoms detached from the red phosphorus segment are gradually located at crystalline sites of black phosphorus. However, some of the atoms are still not properly positioned in the crystalline sites or are not correctly formed covalent bonds to the adjacent phosphorus atoms. According to the observed remarks in the MD simulation, we believe that there are intermediate quasi-crystalline structures, in domains where the number of phosphorus atoms is not enough to form a unit cell. This can disrupt the lattice formation due to the stable energy level of these mid-phase structures. It can also be inferred that during the arrangement of the fifth layer, a percentage of the red phosphorus atoms which are adjacent to the phosphorene began to rearrange in crystallized form (Fig. 6a–o). Furthermore, a division of the RP segment is separated and placed as a structural defect which leads to a relatively defectless structure shown in sub-parts (b) to (h) of Fig. 6a.

### Directional preference in crystallization

In the last step, the simulation results corresponding the computational assessments of directional preference in the crystallization process are presented. A series of sequential MD simulations have been performed on the presented system. The results demonstrated in Fig. 6b, corroborate the formation of the crystal structure after adding the RP atoms in each step.

The table illustrates the step number, the initial and final configurations of the system in each step, respectively. It is worth mentioning that after achieving the final configuration in each step and inserting the new RP segment, the system has been bombarded with hydrogen ions providing the initial configuration for the further step.

By investigating the final arrangement of the atoms in Fig. 6b and according to the accessible time scales in MD simulation, it can be deduced that the tendency of red phosphorus atoms is to be crystalized in the armchair direction rather than the zigzag one. Consequently, we believe that a kind of directional preference in the arrangement of the RP atoms is observable. Moreover, the video of this study presenting the formation of covalent bonds in each step and between monolayer black phosphorus and the red phosphorus atoms has been shown as a supplementary file. Considering this investigation along with the fact that the hopping rate of phosphorene monovacancies along the armchair direction is around 3 orders lower compared to the zigzag one, the possible atomic vacancies formed during the plasma treatment, most likely would not move throughout the structure. In other words, the possibility of extending defect sites will decrease according to the fact that the vacancies extend far along the zigzag direction, however they get attenuated rapidly along the armchair direction^50^.

## Conclusion

We have successfully developed a facile and ultra-fast technique to realize high quality few-layer phosphorene sheets through a rapid phase transition from red phosphorus to black one directly on silicon substrates. Unlike the available methods, this bottom-up technique does not need bulk black phosphorus and has bypassed two inevitable steps in previous approaches, i.e. exfoliation and transfer. It is initiated using a deposited red phosphorus layer on silicon substrates followed by a sequential hydrogen plasma treatment in a plasma reactor to drive a phase transition from amorphous structure to a crystal one. Since the sequential procedure consists of thinning and crystallization steps consecutively, the layer not only crystallizes, but the primary thickness would significantly decrease and that is the key of eliminating exfoliation and further transfer to a second desired substrate. Besides, after the complete phase transition, a transistor with a mobility of ~ 105 cm^2^/Vs and the on/off current ratio of ~ 10^2^ has been fabricated. These ultra-fast fabricated FETs can be mass-produced to be employed especially for the various non-aqueous sensing. Here, by applying a low gate voltage (− 2.5 V), the range of 0.07 to 0.60 mg/ml of the L-Cys has been distinguishably detected while the same response has not observed in positive gates indicating the gate-controlled sensitivity of phosphorene layer to the L-Cys solution.

Furthermore, and to complete the survey by means of the simulation study, the formation process of a monolayer phosphorene from detached segments of red phosphorus through the high-energy impacts of hydrogen ions has been investigated using molecular dynamics simulation. Computational assessments made in this stage aims to implement the process of spreading crystal sites as similar as possible to the experimental method using the computational techniques available in the MD simulation. Analyzing the results achieved from the MD simulation shows a rearrangement in the amorphous structure of the red phosphorus through the expansion of crystal sites and the gradual transformation of the structure into a crystalized monolayer. Moreover, the assessments made to identify the preferred direction of the crystallization indicating a significant tendency to form the crystal structure in the armchair direction compared to the zigzag one. The process conducted in this study can be employed as a paradigmatic framework for implementing molecular dynamics simulation together with experimental studies of crystallization considering the effects of sequential hydrogen plasma treatment. According to the failure of CVD method in realizing large area sheets on silicon wafers, we believe this facile method can be considered as a worthy substitution to form large area phosphorene sheets directly on silicon substrate. Moreover, the capability of ultra-fast and cost-effective production of high quality phosphorene layers can candidate this approach for mass production employing in different types of device fabrication, especially disposable sensors.

## Supplementary Information

Supplementary Video 1.
